# Assessing the impact of a family nurse‐led intervention on young mothers’ references to internal states

**DOI:** 10.1002/imhj.21849

**Published:** 2020-02-11

**Authors:** Amy L. Paine, Rebecca Cannings‐John, Susan Channon, Fiona Lugg‐Widger, Cerith S. Waters, Michael Robling

**Affiliations:** ^1^ Cardiff University Centre for Human Developmental Science School of Psychology, Cardiff University Cardiff UK; ^2^ Centre for Trials Research Cardiff University Cardiff UK

**Keywords:** family nurse partnership, mother–child interaction, randomized‐controlled trial, young motherhood

## Abstract

A mother's propensity to refer to internal states during mother–child interactions is important for her child's developing social understanding. However, adolescent mothers are less likely to reference internal states when interacting with their children. We investigated whether young mothers’ references to internal states are promoted by the Family Nurse Partnership (FNP) intervention, an intensive home‐visiting programme designed to support adolescent mothers in England. We also investigated family, maternal, and child factors associated with young mothers’ references to inner states during interactions with their children. Adolescent mothers (*n *= 483, aged ≤ 19 years when recruited in pregnancy) and their children participated in an observational substudy of a randomized controlled trial investigating the impact of FNP compared to usual care. Mother–child dyads were video‐recorded during free play, and mothers’ speech was coded for use of internal state language (references to cognitions, desires, emotions, intentions, preferences, physiology, and perception). We found no differences in mothers’ use of internal state language between the FNP and usual care groups. A sample‐wide investigation identified that other features of mothers’ language and relationship status with the child's father were associated with internal state language use. Findings are discussed with reference to targeted interventions and implications for future research.

## RESUMEN

La tendencia de la madre a referirse a los estados internos durante las interacciones madre‐niño es importante para el desarrollo social cognitivo de su niño. Sin embargo, las madres adolescentes están menos propensas a referirse a los estados internos cuando interactúan con sus niños. Investigamos si la tendencia de las madres jóvenes a referirse a estados internos es promovida por la intervención de Asociación entre Familia y Enfermera (FNP), un programa intensivo de visitas a casa diseñado para apoyar a madres adolescentes en el Reino Unido. También investigamos factores de familia, maternos y del niño asociados con las interacciones de las jóvenes madres con sus niños. Las madres adolescentes (n = 483, edad ≤ 19 años) y sus niños participaron en un sub‐estudio de observación, un ensayo controlado al azar que investigaba el impacto de FNP comparado con el cuidado usual. Las díadas madre‐niño fueron grabadas en video durante el juego libre, y se codificó el habla de las madres en cuanto al uso del lenguaje de estados internos (referencias a cogniciones, deseos, emociones, intenciones, preferencias, fisiología y percepción). No encontramos diferencias en cuanto al uso del lenguaje de estados internos en las madres entre la FNP y los grupos de cuidado usual. Una muestra de investigación amplia identificó que otras características del lenguaje de las madres y condiciones de la relación con el padre del niño estaban asociadas con el uso del lenguaje de estados internos. Los resultados se discuten con referencia a intervenciones elegidas como objetivo e implicaciones para la futura investigación.

## RÉSUMÉ

La propension d'une mère à faire référence à ses états internes durant les interactions mère‐enfant est importante pour le développement social et cognitif de son enfant. Cependant, les mères adolescentes sont moins à même de faire référence à leurs états internes dans leur interaction avec leurs enfants. Notre étude a porté sur si la propension des jeunes mères à faire référence à leurs états internes est promue par l'intervention Partenariat Famille Infirmières (en anglais Family Nurse Partnership, soit FNP), un programme intensif de visite à domicile conçu afin de soutenir les mères adolescentes au Royaume Uni. Nous avons également étudié les facteurs familiaux, maternels, et liés aux enfants, liés aux interactions des jeunes mères avec leurs enfants. Les mères adolescentes (n = 483, âgés ≤ 19 ans) et leurs enfants ont participé à une sous‐étude observationnelle d'un essai contrôlé randomisé portant sur l'impact de la FNP comparé aux soins normaux. Les dyades mères‐enfant ont été filmées durant un jeu libre et les paroles des mères ont été codées pour son utilisation de langage d’état interne (références aux cognitions, désirs, émotions, intentions, préférences, physiologie et perception). Nous n'avons trouvé aucune différence dans l'utilisation des mères du langage d’état interne entre le groupe FNP et les groupes de contrôle. Une étude de l’échantillon a révélé que d'autres traits du langage des mères et du statut de la relation avec le père de l'enfant étaient liés à l'utilisation de langage de l’état interne. Les résultats sont discutés par rapport aux interventions ciblées et aux implications pour les recherches futures.

## ZUSAMMENFASSUNG

Beurteilung der Wirkungen einer von Familienpflegekräften geleiteten Intervention zur Bezugnahme junger Mütter auf innere Zustände

Inwiefern sich eine Mutter bei den Interaktionen mit ihrem Kind auf innere Zustände bezieht, ist wichtig für die sozial‐kognitive Entwicklung des Kindes. Bei adoleszenten Müttern ist es jedoch weniger wahrscheinlich, dass sie bei der Interaktion mit ihren Kindern auf innere Zustände bezugnehmen. Wir haben untersucht, ob durch die Familienpflegekraft‐Partnerschaftsintervention (Family Nurse Partnership; FNP), ein intensives Hausbesuchsprogramm zur Unterstützung jugendlicher Mütter in Großbritannien, die Bezugnahme auf innere Zustände bei jungen Müttern gefördert werden kann. Ebenfalls untersuchten wir familiäre, mütterliche und kindliche Faktoren die mit der Interaktion junger Mütter mit ihren Kindern assoziiert sind. Die adoleszenten Mütter (n = 483, Alter ≤ 19 Jahre) und ihre Kinder nahmen an einer randomisierten kontrollierten Beobachtungsstudie teil, in der die Wirkung von FNP im Vergleich zur üblichen Betreuung untersucht wurde. Die Mutter‐Kind‐Dyaden wurden während eines freien Spiels auf Video aufgezeichnet und die Sprache der Mütter wurde hinsichtlich ihrer Bezüge auf interne Zustände codiert (Hinweise auf Kognitionen, Wünsche, Emotionen, Absichten, Vorlieben, Physiologie und Wahrnehmung). Zwischen der FNP‐Gruppe und der Gruppe mit der üblichen Betreuung konnten hinsichtlich der Bezugnahme auf innere Zustände keine Unterschiede beobachtet werden. Eine stichprobenweite Untersuchung ergab, dass andere sprachliche Merkmale der Mutter und der Beziehungsstatus zum Kindesvater mit dem Gebrauch von Bezügen auf innere Zustände zusammenhingen. Die Ergebnisse werden hinsichtlich gezielter Interventionen und Implikationen für zukünftige Forschung diskutiert.

## 抄録

**若年母親の内的状態に関する言及への家族看護師主導介入の影響を評価する**

母子相互交流時における母親の内的状態について言及する傾向は、乳幼児の社会的認知の発達に重要である。しかしながら、若年母親は自分の子どもとの相互交流時に内的状態について言及する傾向が弱い。我々は、若年母親が内的状態を言及する傾向が、ナース・ファミリー・パートナーシップ(FNP)介入−それは、英国における若年母親を支援するために考案された集中的な家庭訪問プログラム−によって促進されるかどうかについて研究した。また、家族、母親と乳幼児それぞれの、若年母親の自分の子どもとの相互交流に関連する要素について研究した。若年母親(n = 483, 年齢 ≤ 19歳)とその子どもは普通のケアに比較してFNPの影響を調査するランダム化比較試験の観察下位研究に参加した。母子親子は自由遊び時を録画され、母親の発言は内的状態言語(認知、欲望、情緒、意図、好み、生理、知覚の言及)の使用についてコード化された。FNPと普通のケア群との間の母親の内的言語の使用に差異はなかった。サンプル全体の調査は母親の内的言語とは違う言語と子どもの父親との関係性は、内的状態言語の使用に関連していることが同定された。 結果は対象を絞った介入についての言及と今後の研究での予想される結果について討論している。

## 摘要

母亲在母子互动过程中倾向于提及内在状态, 这对孩子的社会认知发展很重要。但是, 未成年母亲与其子女互动时却不太可能提及内在状态。我们调查了“家庭护士伙伴关系计划 (FNP) ”的干预是否促进了未成年母亲提及内在状态的倾向, 该干预是一项旨在支持英国未成年母亲的密集的家访计划。我们还调查了与年轻母亲及其孩子互动有关的家庭、母亲和儿童方面的因素。未成年母亲 (n = 483, 年龄≤19岁) 及其子女参加了一项观察性的子研究, 这是一项随机对照试验, 调查FNP与常规护理相比的影响。在自由活动期间, 母子二人组被录制了视频, 母亲的谈话被编码, 以记录其使用内在状态的语言 (涉及到认知、欲望、情绪、意图、偏好、生理和感知) 。我们发现FNP组和常规护理组的母亲在使用内在状态语言方面没有差异。一项抽样调查显示, 母亲语言的其他特征以及与孩子父亲的关系状态都和内在状态语言的使用有关。研究结果将参考有针对性的干预措施和对未来研究的影响进行讨论。

## ملخص

**تقييم أثر تدخل عائلي قائم على التمريض على إشارات الأمهات الشابات إلى حالاتهم النفسية الداخلية **

يعتبر ميل الأم للإشارة إلى الحالات النفسية الداخلية أثناء التفاعلات بين الأم والطفل مهم للنمو المعرفي الاجتماعي لطفلها. ومع ذلك، فإن الأمهات المراهقات أقل احتمالا للإشارة إلى الحالات الداخلية عند التفاعل مع أطفالهن. لقد حققنا فيما إذا كان ميل الأمهات الشابات إلى الإشارة إلى الحالات النفسية الداخلية يزداد من خلال تدخل شراكة العائلة والتمريض (FNP)، وهو برنامج مكثف للزيارات المنزلية يهدف إلى دعم الأمهات المراهقات في المملكة المتحدة. كما قمنا بتحري العوامل الأسرية والأمومية والعوامل المرتبطة بتفاعل الأمهات الشابات مع أطفالهن. شاركت الأمهات المراهقات (العدد = 483، البالغات من العمر ≤ 19 سنة) وأطفالهن في دراسة فرعية قائمة على الملاحظة وهي تجربة معشاة ذات شواهد تبحث في تأثير تدخل شراكة العائلة والتمريض FNP مقارنة بالرعاية المعتادة. تم تسجيل فيديوهات لثنائيات الأم والطفل الفيديو خلال اللعب الحر، وتم ترميز خطاب الأمهات في استخدام لغة الحالة الداخلية (إشارات إلى المعرفة، والرغبات، والعواطف، والنوايا، والتفضيلات، والعوامل الفسيولوجية والإدراك). لم نجد أي اختلافات في استخدام الأمهات للغة الحالة الداخلية بين FNP ومجموعات الرعاية المعتادة. وقد تبين من تحقيق على نطاق العينة أن السمات الأخرى للغة الأمهات وحالة العلاقة مع والد الطفل ترتبط باستخدام لغة الحالة الداخلية. وتناقش النتائج بالإشارة إلى التدخلات المستهدفة وتطبيقات البحوث المستقبلية.

Adolescent mothers face numerous individual, social, and economic challenges that place them at increased risk for less optimal parenting practices (Firk, Konrad, Herpertz‐Dahlmann, Scharke, & Dahmen, 2018). Several studies demonstrate that adolescent mothers are at risk for providing a less stimulating language environment for their children than adult mothers (McDonald‐Culp, Osofsky, & O'Brien, 1996; Whitman, Borkowski, Keogh, & Weed, 2001). One aspect of maternal language associated with adolescent motherhood is *internal state language*; a feature of maternal language related to children's social cognitive development (Demers, Bernier, Tarabulsy, & Provost, 2010; Tompkins, Benigno, Lee, & Wright, 2018). Given that the cumulative effects of adversity associated with young motherhood and suboptimal caregiving environments can place children on a trajectory for later sociocognitive problems (Riva Crugnola, Ierardi, Gazzotti, & Albizzati, 2014), interventions that foster a mother's mind‐related discourse with her child may be one avenue by which her child's outcomes are improved (Aram, Fine, & Ziv, 2013). However, to our knowledge, no studies have investigated the impact of interventions available to adolescent mothers on their production of internal state language during mother–child interactions. Therefore, in the present study, we explored whether one of the efforts of the Government in England to support vulnerable young mothers and their first‐born children through specialist home visiting (the *Family Nurse Partnership*; FNP) increases mothers’ propensity to discuss internal states during interactions with their 24‐month‐old children.

## 1 PROMOTING ADOLESCENT MOTHERS’ REFERENCES TO INTERNAL STATES

A mother's proclivity to discuss internal states with her child is an important marker of maternal behavior within mother–child interactions and of children's developmental outcomes. Mothers’ internal state language within interactions with their children, such as references to cognitions (thoughts and knowledge), emotions, and desires, is positively associated with other markers of positive maternal behaviors, such as the quantity of maternal speech (e.g., Roberts et al., 2013). Relatedly, maternal mind‐mindedness―a mother's explicit use of mental state language to comment appropriately on her infant's mind―is associated with attachment‐related differences (Meins, Fernyhough, Fradley, & Tuckey, 2001). Mothers’ internal state language also fosters children's social understanding skills (Tompkins et al., 2018), which in turn, are related to numerous positive child outcomes. For example, children with better social understanding skills have more positive interactions with peers (Dunn & Cutting, 1999), improved teacher ratings of social competence (Lalonde & Chandler, 1995), and do well academically (Lecce, Caputi, & Hughes, 2011).

Generally, compared to adult mothers, adolescent mothers’ interactions with their children are less conducive to optimal early cognitive and social development (Oxford & Spieker, 2006). Adolescent motherhood is associated with a constellation of risk factors that may impact childrearing capabilities, such as limited educational opportunities, greater economic instability, and risk for psychological problems (Jaffee, Caspi, Moffitt, Belsky, & Silva, 2001). Indeed, adolescent mothers tend to provide less verbal stimulation, positive affective speech, warm affection, and sensitivity (Garner, Rennie, & Miner, 1996; Keown, Woodward, & Field, 2001), and demonstrate more negative behaviors (e.g., rough handling) when interacting with their children (McDonald‐Culp et al., 1996). More recent evidence also shows that compared to adult mothers, adolescent mothers refer to emotional and mental inner states less often with their children in infancy and toddlerhood (Riva Crugnola, Ierardi, & Canevini, 2018). Discussing internal states with their children may be challenging for adolescent mothers given their own level of maturity, considering the late maturation of frontal lobe circuity in adolescence (Giedd, 2008), as well as their possible lack of knowledge about caregiving and tendency to reside in an adverse environment (Jaffee et al., 2001).

Given increasing concern regarding the outcomes of young and vulnerable mothers and their children, numerous parenting interventions are designed to improve outcomes for young mothers and their children (Guttentag et al., 2014; McGowan et al., 2008; Olds et al., 2002). Programmes that place emphasis on improving parenting skills can be effective in improving children's cognitive outcomes, as the relationship between intervention effects and positive child outcomes can be mediated by improvements in the quality of mother–child interactions (Baudry, Tarabulsy, Atkinson, Pearson, & St‐Pierre, 2017). Evidence suggests that mothers’ references to internal states can be promoted via targeted intervention (Aram et al., 2013), and recently, short‐term attachment‐based interventions have been shown to facilitate mind‐mindedness in adolescent and young mothers (Riva Crugnola, Ierardi, Peruta, Moiloli, & Albizzati, 2019). However, few studies have investigated the impact of attachment‐based home visiting programmes on mothers’ capacity to reflect on inner states.

The FNP programme is one such home visiting intervention available to adolescent first‐time mothers residing in England. FNP was introduced to England in 2007 as an adaptation of the US‐based *Nurse Family Partnership* (NFP) programme (Olds et al., 2002) and provides an intensive home visiting service for first‐time teenage mothers from pregnancy to 24 months postpartum (Robling et al., 2016). The aim of FNP is to promote sensitive and competent caregiving by educating mothers on child development and by modeling sensitive parent–child interactions. Studies that have assessed the impact of NFP and FNP on mother–child interactions have reported mixed findings: Nurse‐visited mother–child dyads interacted more responsively than their control group counterparts, as did paraprofessional‐visited mother–child dyads, but only when the mother had low psychological resources (Olds et al., 2002). Four years later, paraprofessional‐visited mother–child dyads continued to show more sensitive and responsive interactions compared to controls (Olds et al., 2004). However, other studies have found no effect of FNP on mother–child sensitivity (Robling et al., 2016).

FNP is grounded in attachment theory, which conceptualizes the child as an independent person with their own thoughts, feelings, and desires, and emphasizes the importance of maternal reflection and communication of internal states (Ainsworth, Blehar, Waters, & Wall, 2014). However, maternal references to inner states are rarely measured as an outcome of attachment‐based home visiting interventions. In one exception, an evaluation of an adaptation of NFP; an interdisciplinary, mentalization‐based intervention within a home visiting service, found that maternal *reflective functioning* (or mentalization)―a mother's ability to envision and reflect on the internal and psychological world of herself and others―was improved over the course of the intervention for mothers whose reflective functioning was very low at enrollment to the study (Sadler et al., 2013). It has been suggested that behaviorally based measures of mothers’ propensity to refer to inner states within mother–child interactions may be a more appropriate approach (Riva Crugnola et al., 2019; Sadler et al., 2013). Therefore, we investigated whether young mothers who were recipients of FNP produced more references to internal states within interactions with their toddlers than their counterparts who experienced usual health and social care.

## 2 FAMILY, MATERNAL, AND CHILD CHARACTERISTICS ASSOCIATED WITH ADOLESCENT MOTHERS’ INTERNAL STATE LANGUAGE

Few studies have investigated adolescent mothers’ references to internal states (Demers et al., 2010), and as such, the sources of individual differences in young mothers’ propensity to refer to internal states are relatively unknown. Although family and maternal characteristics are typically included as covariates in studies of mothers’ references to internal states (Dunn, Brown, Slomkowski, Tesla, & Youngblade, 1991), to date, these investigations have taken place within the context of low‐risk samples. Given that determinants of parenting differ between adolescent and adult mothers (Shapiro & Mangelsford, 1994), it is likely that family, maternal, and child characteristics typically associated with adult mothers’ propensity to use internal state language differ among adolescent mothers (Demers et al., 2010).

**Statement of relevance to field**

This study provides evidence of a lack of programme effectiveness on young mothers’ internal state language; a marker of positive maternal behavior that fosters children's social understanding skills.

**Key Findings and Implications**

**Finding 1**: We detected no differences in young mothers’ use of internal state language during interactions with their 24‐month‐olds between those who received a programme of home visiting (the Family Nurse Partnership, FNP) and those who received usual care alone.

**Implication 1**: Further research is needed to investigate the effectiveness of FNP on other features of mother–child interactions. Longitudinal follow‐up will determine whether benefits of FNP may also arise later in the maternal and child life course.

**Finding 2**: Mothers who had a higher mean length of utterance (MLU) and identified as being friends with the father of their child over having no relationship produced more references to internal states.

**Implication 2**: Mothers who use more internal state language may be better able to maintain positive relationships with the father of their child, or, the positive effects of father involvement may result in more internal state references.

Family sociodemographic risk factors, such as maternal education and social class, are known correlates of mothers’ production of internal state language for low‐risk mothers (Dunn et al., 1991; Paine, Hashmi, Roberts, Fyfield, & Hay, 2019). Given that we may expect different risk factors to be associated with adolescent mothers’ internal state language, we widened the investigation to additional sociodemographic and maternal risk factors, such as number of people living in the home and mothers’ relationship status with the child's father. Having two parents in the home, a higher income, less crowding, and more stability may result in less parent stress and more time to discuss internal states with children (Pears & Moses, 2003).

It is well established that the socioeconomic hardship associated with young motherhood places adolescent mothers at a higher risk for mental health and behavioral problems (Romano, Zoccolillo, & Paquette, 2006). Adolescent mothers have a high incidence of depression (reported rates of 30–59%), a higher risk for postpartum depression, and are more likely to have a history of behavior problems, all of which are known to impact negatively on mother–child interactions (Beers & Hollo, 2009; Jaffee, Belsky, Harrington, Caspi, & Moffitt, 2006; Kim‐Cohen, Caspi, Rutter, Tomás, & Moffitt, 2006). For example, depressed mothers speak less frequently, with less affective and informative speech, and are less likely to use motherese (Bettes, 1988; Herrera, Reissland, & Shepherd, 2004), all of which are linked to a mother's propensity to reflect on her child's emotional and cognitive states (Saint‐George et al., 2013). More specifically, maternal depression is associated with a reduction in a mother's ability to comment appropriately on the internal states of her infant (Pawlby et al., 2010).

We also investigated child characteristics that may be associated with mothers’ production of internal state language. According to transactional models of parenting and child development (Sameroff, 2009), characteristics of the child elicit certain behavior from their parent; in the case of internal state language, children who are female may elicit more internal state language from their mothers (Barnett, Gustafsson, Deng, Mills‐Koonce, & Cox, 2012; Leaper, Anderson, & Sanders, 1998).

## 3 SUMMARY OF AIMS AND HYPOTHESES

We aimed to investigate the impact of a licensed intensive home visiting intervention (FNP) designed for first‐time adolescent mothers on mothers’ use of internal state language during interactions with their toddlers. We explored observational data from the Building Blocks randomized controlled trial designed to investigate the impact of FNP in England compared to universally available care (Robling et al., 2016). We harnessed data from a subsample of mothers and children who were observed when the child was 24 months old, and coded for mothers’ use of internal state language. We hypothesized that mothers in the FNP group would produce more references to internal states than those in the usual care group. Finally, we conducted an exploratory investigation into the family, maternal, and child‐related sources of individual differences in adolescent mothers’ propensity to use internal state language when interacting with their firstborn child.

## 4 METHOD

### 4.1 Design

The original Building Blocks trial was a pragmatic, nonblinded, randomized controlled, parallel‐group trial (see Owen‐Jones et al., 2013 for protocol), designed to compare usual care (through primary care public health and social care services) plus FNP (FNP group) to usual care alone (usual care group). The original trial took place in 18 sites across England, where local partnerships, including primary and secondary National Health Service (NHS) organizations, and local authorities were established to provide FNP. The present study focuses on a subsample of mothers and their children who provided observational data within the original trial, forming the Baby and Adult Building Blocks Language Evaluation (BABBLE). Multicenter ethical approval was granted by the Research Ethics Committee for Wales (ref. no. 09/MRE09/8). Site‐specific approval has been granted at all participating Primary Care and Acute Trusts.

### 4.2 Participants

In the original trial, 1,645 nulliparous women under the age of 19 and living within a catchment area of an FNP team were randomly assigned to FNP (*n *= 823) or to usual care (*n *= 822). Randomization was stratified by site and minimized by gestation at recruitment (< 16 vs. > 16 weeks), smoking status at recruitment (yes vs. no), and preferred language of data collection (English vs. non‐English) with a probability of 0.80. In the original trial, data were collected at baseline (prior to intervention allocation), late pregnancy, and at 6, 12, 18, and 24 months postpartum (see Robling et al., 2016). The present study comprises a subsample of 483/1,154 (41.9%) mother–child dyads who took part in the 24‐month follow‐up assessment (child mean age 24.63, *SD* = 1.38, range 22–33 months, 48.9% females), who provided additional consent to be video‐recorded for 3 min during free play with their firstborn child.

### 4.3 Procedure

#### 4.3.1 Intervention

FNP is an evidence‐based nurse‐led intensive home visiting programme for women expecting their first baby. FNP was originally developed in the United States (University of Colorado, Denver) as the NFP (Olds, Henderson, Chamberlin, & Tatelbaum, 1986), and is designed to improve perinatal outcomes, child health and developmental outcomes, and parent economic self‐sufficiency. The programme is based on three theoretical approaches: attachment theory (Bowlby, 1982); ecological theory (Bronfenbrenner, 1979); and self‐efficacy theory (Bandura, 1977). Trained Family Nurses (FNs) conduct home visits starting from early pregnancy until the first child reaches 24 months of age. Although the number of visits is determined by individual need, families can receive a maximum of 64 scheduled visits: 14 during pregnancy; 28 during infancy (0–12 months postpartum); and 22 during toddlerhood (13–24 months postpartum). In the programme, FNs cover content, including personal and environmental health, life course development, maternal role, family and friends, and access to health and Social Services (Table 1). Alongside FNP, FNs provide usual maternity services during the pregnancy and neonatal period, and from 1 to 24 months postnatally, they undertake the role of the Health Visitor (also delivering the Healthy Child Programme).

**Table 1:** Content of home visits and target percentage of time FNs spent in each domain

Phase	Personal health (%)	Maternal role (%)	Friends and family (%)	Life course (%)	Environmental health (%)
Pregnancy	35–40	23–25	10–15	10–15	7–10
Infancy	14–20	45–50	10–15	10–15	7–10
Toddlerhood	10–15	40–45	10–15	18–20	7–10

#### 4.3.2 Usual care

Participants in the usual care arm received care from their local maternity services, as well as their postnatal midwifery care and care from existing child health services available locally, including an allocated Health Visitor.

#### 4.3.3 Data collection

Routine data, such as antenatal, birth, and neonatal data, were collected from maternity records. Secondary care data were collected via NHS Digital. Primary care data were collected directly from general practitioner (GP) records. A baseline assessment prior to allocation at 24 months was conducted by field‐based researchers using computer‐assisted personal interviews (CAPIs). Computer‐assisted telephone interviews were conducted at late pregnancy and 6, 12, and 18 months postpartum by office‐based researchers who were blind to the trial arm. Participants were not blind to the intervention. Although 24‐month CAPIs were completed by researchers not blinded to trial arm, they occurred independent of service delivery.

For those families who provided additional consent at the 24‐month assessment, mother–child dyads were video recorded as they interacted during a 3 min free play session in the home. Each mother–child dyad was provided with a standardized set of toys (stacking cups, bells, a stuffed Winnie the Pooh/Tigger, and a wind‐up car) and a blanket to play on. Mother–child dyads were asked to play whichever way they liked, and could also use their own toys or books as they wished. Similar durations of parent–child interaction and free play with toys have been shown to be a rich context for the study of internal state language (see Paine et al., 2019; Roberts et al., 2013).

#### 4.3.4 Transcripts

All meaningful speech by mothers was transcribed verbatim from the video records by research assistants who were blind to the trial arm. Research assistants initially transcribed all maternal utterances, which was defined as speech bounded by grammatical closure, a transition in speaker, or by a pause or change in intonational pattern (Suskind et al., 2016). Transcript agreement was established for a sample of 24 (5%) of cases with agreement at 91.4% for maternal speech. Of those who provided audio‐visual data, seven dyads were excluded (five did not take place in English and two had technical errors), yielding a final subsample of 476/483 (98.6%) (FNP = 243, usual care = 233) mother–child dyads.

### 4.4 Measures

#### 4.4.1 Mothers’ mean length of utterance

Mothers’ mean length of utterance (*MLU*) was coded according to Brown's (1973) classic recommendations for calculating MLU in morphemes. A 15% random subsample of cases (71/476) was coded by a second research assistant, blind to trial arm, to establish reliability for calculations of maternal MLU (*ICC* = .99).

#### 4.4.2 Mothers’ references to internal states

All utterances were coded for mothers’ *references to internal states*, using an appropriate and reliable scheme for coding mothers’ internal state language during interactions with their children across a wide age range and within similarly concentrated time periods (Paine et al., 2019). A mother's references to her own, her child's, or any toy character or other present/nonpresent individual were coded. Internal state language was divided into seven categories, including comments about: (a) *perception* of an object using one of the five senses (“Can you hear it?” “What are you looking for?”; (b) *physiology*, physical states, and sensations, (“Oh, you hurt your leg” “Tired, aren't you?”); (c) *preferences*, positive or negative judgements about an object, action, or experience (“Do you like these?” “Is this your favourite?”); (d) goal‐directed and future *intentions* (“You gonna give Pooh a cuddle?” “You gonna do peek‐a‐boo?”); (e) *desire* for an object, action, or experience (“Do you want to count with mummy?” “Wanna play with this one?”); (f) *emotions* (“Who's grumpy?” “We're very happy with these, aren't we?”); (g) *cognitions*, beliefs and knowledge (“You know what to do with these, don't you?” “Can you remember?”). An independent observer coded mothers’ internal state language for 71/476 cases (15% of transcripts), where excellent inter‐rater reliability was established (*ICC*s ranged from .93 to 1.00). Instances of mothers’ internal state language were counted to form a frequency count mothers’ overall total frequency of references to internal states, and for frequency counts for each category.

#### 4.4.3 Sociodemographic characteristics

Variables included: (a) *maternal age* at recruitment; in addition to sociodemographic characteristics at the 24‐month assessment: (b) not in education, employment, or training (*NEET status*); (c) *number of people living in the home;* (d) *relationship status with the child's father*; and (e) *language spoken in the home* (English or English and other). At recruitment, an overall measure of deprivation was recorded using the *Index of Multiple Deprivation* (IMD; Wilkinson, Sniehotta, & Michie, 2011).

#### 4.4.4 Maternal psychological distress

Mothers’ *psychological distress* at 24 months was assessed using the Kessler Psychological Distress Scale, a 10‐item screening scale that discriminates with precision DSM‐IV cases from noncases (Kessler et al., 2002) and was scored between 10 and 50 (with higher scores indicating more distress).

#### 4.4.5 Postnatal depression

*Postnatal depression (PND)* was assessed at the 6‐month follow‐up assessment using the Edinburgh Postnatal Depression Scale (EPDS; Cox, Holden, & Sagovsky, 1987). In this 10‐item scale, symptoms of PND are scored between 0 and 30 (with higher scores indicating more symptoms).

#### 4.4.6 Antisocial behavior

Mothers’ *history of antisocial behavior* at baseline was assessed using an adapted version of Zoccolillo's (2004) measure of maternal and paternal antisocial disorder. In the original measure, items differed according to participant gender. In the present study, mothers were asked all (male and female) items. These included whether the mother had ever: (a) stolen; (b) got into fights; (c) had trouble with police/been arrested; (d) engaged in truancy; (e) run away overnight; and (f) ever been suspended/expelled/excluded. These items were totaled to produce a summary score between 0 and 6 (higher scores indicating more antisocial behavior).

### 4.5 Statistical analysis

Total use of internal state language and individual categories showed overdispersion of the count data, and therefore differences in mothers’ internal state language by group were analyzed using negative binomial models. Data preparation and analysis was conducted using SPSS version 20 and Stata version 13. All models were adjusted for minimization variables and by site. Internal state categories with high zero counts (emotion and physiology) were analyzed using zero‐inflated negative binomial models. In this approach, two outcomes are produced: the first assumes a logistic regression model (count vs. no count) and the second assumes a negative binomial distribution for the counts portion only (Atkins & Gallop, 2007). Negative binomial analyses were presented as incidence rate ratios (*IRR*s), and logistic regression portions of zero‐inflated models were presented as odds ratios, comparing the odds of internal state language production occurring within the FNP group compared to the usual care group.

Following the investigation of group differences, the final aim of the present study was to investigate predictors of young mothers’ propensity to use internal state language. The BABBLE subsample comprises 483 mother–child dyads. To test 20 predictors (assuming medium effect sizes, alpha = 0.05, power = 80%) 159+ individuals are required, suggesting adequate power for the proposed analyses examining predictors of mothers’ internal state language. Sample‐wide predictors of mothers’ overall frequency of internal state language were first examined on the univariate level using negative binomial models. Predictors that reached 10% significance level in the univariate tests were followed up with multivariate negative binomial regression. Negative binomial analyses were presented as *IRR*s.

## 5 RESULTS

### 5.1 Description of sample

Of the 483 mother–child dyads who took part in BABBLE, 246 dyads were allocated to FNP (50.9%) and 237 to usual care (49.1%). The sample was balanced according to baseline sociodemographic variables between the FNP and usual care groups (all *p*s > .05). Descriptive data for the BABBLE sample are presented in Table 2. When compared to the original Building Blocks trial, the BABBLE sample was significantly different in terms of maternal ethnicity (with fewer participants of black backgrounds), education (fewer with no qualifications), and more spoke only English in the home (all *p* < .05, available under request). The participants in the BABBLE sample assigned to the FNP arm also received significantly more FN visits than those in the FNP arm of the original trial, mean difference = 5.87, 95% CI [3.53‐8.22], *p* < .001.

**Table 2:** Descriptive statistics for predictors of interest

	BABBLE sample (*N* = 476)
Maternal age at recruitment (years)	
Mean *(SD)*	17.91 (1.22)
Range	13.82–19.98
NEET status at 24 months *N* (%)	
Yes	307 (64.5)
No	169 (35.5)
Number of people living in the home at 24 months	
Mean *(SD)*	1.03 (1.31)
Range	0–7
Relationship with child's father at 24 months*N (%)	
Married	15 (3.2)
Separated/divorced	25 (5.3)
Closely involved/boyfriend	208 (43.7)
Just friends	91 (19.1)
Not in any relationship	136 (28.6)
Missing	1 (0.2)
Language spoken in the home^ *N* (%)	
English only	467 (98.1)
English and another language	9 (1.9)
IMD score at baseline	
Mean *(SD)*	38.52 (17.96)
Range	3.15–82.00
Psychological distress at 24 months	
Mean *(SD)*	17.10 (1.27)
Range	10–43
Postnatal depression at 6 months	
Mean *(SD)*	6.71 (5.05)
Range	0–24
History of antisocial behavior at baseline	
Mean *(SD)*	2.23 (1.72)
Range	0–6
Mother MLU at 24‐month interaction	
Mean *(SD)*	3.01 (0.57)
Range	1.44–4.75

*Note*. One in sample was divorced, so merged with “separated.” Higher IMD score indicates more deprivation. Mean IMD score for England in 2010 was 21.67 (Wilkinson et al., 2011).

IMD, Index of Multiple Deprivation; MLU, mean length of utterance (in morphemes); NEET, not in education, employment, or training.

Descriptive data for mothers’ use of internal state language within the BABBLE sample are presented in Table 3. Within the BABBLE sample who had mother–child interaction data available, the majority of mothers referred to at least one internal state within the interaction with their child, 426/476 (89.5%). Overall, mothers referred most often to desires, intentions, and cognitions.

**Table 3:** Description of internal state language for BABBLE sample and between FNP and usual care groups

Categories of internal state language	FNP *n *= 243	Usual care *n *= 233	BABBLE sample *N* = 476
Perception			
Mean *(SD)*	0.40 (1.08)	0.30 (0.81)	0.35 (0.96)
Range	0.00–9.00	0.00–8.00	0.00–9.00
Physiology			
Mean *(SD)*	0.18 (0.82)	0.11 (0.55)	0.15 (0.70)
Range	0.00–7.00	0.00–5.00	0.00–7.00
No count *N* (%)	226 (93.00)	220 (94.40)	446 (93.70)
Count *N* (%)	17 (7.00)	13 (5.60)	30 (6.30)
In those with a count, mean *(SD)*	2.59 (1.91)	2.00 (1.35)	2.33 (1.69)
Preference			
Mean *(SD)*	0.26 (0.66)	0.29 (0.84)	0.28 (0.75)
Range	0.00–4.00	0.00–6.00	0.00–6.00
Intention			
Mean *(SD)*	1.19 (1.58)	1.04 (1.43)	1.12 (1.51)
Range	0.00–10.00	0.00–8.00	0.00–10.00
Desire			
Mean *(SD)*	1.51 (1.84)	1.51 (1.92)	1.51 (1.87)
Range	0.00–10.00	0.00–10.00	0.00–10.00
Emotion			
Mean *(SD)*	0.04 (0.21)	0.03 (0.16)	0.03 (0.19)
Range	0.00–2.00	0.00–1.00	0.00–2.00
No count *N* (%)	235 (96.70)	227 (97.40)	462 (97.10)
Count *N* (%)	8 (3.30)	6 (2.60)	14 (2.90)
In those with a count, mean *(SD)*	1.13 (0.35)	1.00 (0.00)	1.07 (0.27)
Cognition			
Mean *(SD)*	0.60 (1.14)	0.58 (1.17)	0.59 (1.15)
Range	0.00–12.00	0.00–8.00	0.00–12.00
Total internal state language			
Median *(IQR)*	3.00 (1.00–6.00)	3.00 (1.00–5.50)	3.00 (1.00–6.00)
Mean *(SD)*	4.17 (3.69)	3.86 (3.56)	4.02 (3.63)
Range	0.00–26.00	0.00–19.00	0.00–26.00

### 5.2 Mothers’ internal state language between FNP and usual care groups

Descriptive data for mothers’ internal state language by category within the FNP and usual care groups are presented in Table 3. No differences were detected between FNP and usual care groups in the overall frequency of internal state language during the mother–child interaction, adjusted (for minimization variables and site) *IRR* = 1.08, where *IRR* > 1 indicates greater counts of internal state language in the FNP arm, 95% *CI* for *IRR* [0.92–1.27], *p* = .36. Similarly, no differences between study groups were detected for mothers’ individual categories of internal state language (all *p*s > .05).

### 5.3 Individual differences in young mothers’ references to internal states

Factors associated with young mothers’ total references to internal states were investigated within the BABBLE sample. Descriptive statistics for predictors of interest are presented in Table 2. Predictors of interest were first investigated on the univariate level (Table 4). The mothers’ deprivation score and not being in education, employment, or training was associated with fewer internal state references (*p* < .10); mothers in education, employment, or training *M* = 4.59 (*SD *= 3.83), mothers *not* in education, employment, or training *M* = 3.67 (*SD *= 3.47). Mothers’ relationship with the child's father at the time of the 24‐month assessment was associated with their references to internal states, where mothers who were friends with the father (*M* = 4.81, *SD *= 4.47) made more references to inner states than mothers who self‐defined as not being in any relationship with their child's father (*M* = 3.44*, SD *= 3.11). Mother MLU scores were associated with more maternal internal state language, as was child gender, where mothers referred to internal states more to their daughters (*M* = 4.36*, SD *= 3.96) than their sons (*M* = 3.65, *SD *= 3.24).

**Table 4:** Predictors of mothers’ total references to internal states

	*IRR*	95% *CI*	*p*‐value
Maternal age at recruitment	1.01	0.95‐1.08	.75
NEET status at 24 months			
Yes	0.80	0.68‐0.95	.01
No	Reference		
Number of people living in the home at 24 months	1.04	0.98‐1.11	.22
Relationship with child's father at 24 months			
Married	1.40	0.88‐2.22	.16
Separated	1.00	0.68‐1.48	1.00
Closely involved/boyfriend	1.18	0.97‐1.43	.10
Just friends	1.40	1.11‐1.77	.005
Not in any relationship	Reference		
Language spoken in the home			
English and another language	0.77	0.43‐1.35	.36
English only	Reference		
IMD score at baseline	1.00	0.99‐1.00	.09
Psychological distress at 24 months	1.02	0.99‐1.01	.70
Postnatal depression at 6 months	0.99	0.97‐1.01	.44
History of antisocial behavior at baseline	0.99	0.94‐1.04	.66
Mother MLU at 24‐month interaction	2.07	1.80‐2.37	.001
Child gender			
Female	1.20	1.02‐1.40	.03
Male	Reference		

*Note*. The foregoing analyses are on the univariate level. Negative binomial models. *IRR* > 1 indicates greater counts of internal state language. IMD, Index of Multiple Deprivation; MLU, mean length of utterance (in morphemes); NEET, not in education, employment, or training.

These predictors were brought forward into a multivariate analysis (Table 5), which demonstrated that mothers’ relationship status (being friends with the father over no relationship) and mothers’ MLU were significantly associated with mothers’ overall frequency of internal state language, *IRR* = 1.43, 95% *CI* 1.16‐1.77 and *IRR* = 2.03, 95% *CI* 1.77‐2.33, respectively.

**Table 5:** Multivariate analysis of predictors of mothers’ total references to internal states

	*IRR*	95% *CI*	*p*‐value
NEET status at 24 months			
Yes	0.91	0.78‐1.07	.28
No	Reference		
Deprivation score at baseline	0.99	0.99‐1.00	.22
Relationship with baby's father at 24 ms^a^			
Married	1.50	0.99‐2.27	.055
Separated	0.99	0.70‐1.42	.99
Closely involved/boyfriend	1.12	0.94‐1.34	.19
Just friends	1.43	1.16‐1.77	.001
Not in any relationship	Reference		
Child gender			
Female	1.13	0.97‐1.31	.09
Male	Reference		
Mother MLU	2.03	1.77‐2.33	.001

*Note. N *= 471. Negative binomial model. *IRR* > 1 indicates greater counts of internal state language.

IMD, Index of Multiple Deprivation; MLU, mean length of utterance (in morphemes); NEET, not in education, employment, or training.

## 6 DISCUSSION

The children of adolescent mothers are at risk for poorer social and cognitive outcomes compared to children of adult mothers (Riva Crugnola et al., 2014); the relationship between socioeconomic hardship and children's cognitive development is mediated by adolescent mothers’ caregiving practices (Firk et al., 2018). As such, prevention and intervention programmes that focus on improving mother–child interactions may be one avenue whereby children's outcomes can be improved. In this observational study within the context of a randomized controlled trial, we found no statistically significant differences in mothers’ references to internal states within mother–child interaction in the FNP compared to the usual care group. Our investigation of sample‐wide correlates of young mothers’ internal state language indicated that mothers’ MLU and relationship status were associated with the use of internal state language within mothers’ interactions with their toddlers.

It is possible then that young mothers’ discussions about inner states with their children may be better fostered by more targeted intervention strategies. For example, in a low‐socioeconomic status sample, Aram et al. (2013) found that a 4‐week book reading workshop had a positive impact on the number of sociocognitive themes discussed by parents during shared reading. Brief and medium‐term interventions that focus on increasing appropriate mind‐related comments through video‐feedback have led to improvements in the quality of maternal mind‐related comments in adolescent mothers (Riva Crugnola et al., 2019) in addition to clinically referred mothers and mothers with severe mental illness (Schacht et al., 2017; Zeegers et al., 2019). Findings from a recent meta‐analysis also indicate that shorter intervention strategies may be most effective for promoting the cognitive development of children of young mothers; adolescent mothers may find shorter, targeted interventions that supply “take home messages” most helpful and easy to integrate into daily life (Baudry et al., 2017). Indeed, other attachment‐based programmes that use video‐feedback are also shown to be an effective intervention for improving young mothers’ parenting (e.g., Slade, Sadler, & Mayes, 2005).

However, further work is needed to determine the impact of home visiting and attachment‐based programmes on mothers’ references to inner states. It is possible that, where service provision is limited, FNP could be expected to have a greater positive impact on mothers’ internal state language than in settings where usual care itself offers a comprehensive support package. Along a similar vein, FNP may have a greater impact for the most vulnerable mothers. Indeed, in US trials, some short‐term effects of NFP were most evident for the most at‐risk mothers (Olds et al., 1986; 2002), and in Sadler et al. (2013) adapation of NFP, maternal reflective functioning was only improved over the course of the intervention in the most high‐risk mothers. Not only may the original trial represent a less disadvantaged group (Robling et al., 2016), but the sample who volunteered to be recorded in the present observational study may also come from, comparatively, less disadvantage (e.g., fewer had no educational qualifications). Further work investigating the effect of FNP (comparing low to high dosage) among the most vulnerable of young mothers is warranted.

Given that the study groups were balanced in terms of internal state language use, this provided a unique opportunity to explore predictors of young mothers’ propensity to refer to internal states when interacting with their toddlers. Young mothers’ total use of internal state language was positively associated with maternal MLU in morphemes and with mothers’ relationship status with the child's father at the time of the observation. Specifically, mothers who were friends with the father of the child made a higher number of internal state references during the interaction with their children than mothers who self‐defined as having no relationship with their child's biological father. Given that mothers’ propensity to refer to internal states is characterized as a cognitive behavioral trait (Meins, Fernyhough, Arnott, Turner, & Leekam, 2011), it is possible that mothers who tend to use more internal state language are better able to maintain positive relationships with the father of the child. Alternatively, friendship with the father may represent paternal involvement with the child. Consistent father involvement is associated with lower levels of psychological problems in adolescent mothers (Kalil, Ziol‐Guest, & Coley, 2005). As such, positive effects of father involvement may be related to mothers’ increased proclivity to discuss internal states with her child. Given that fathers of children born to adolescent mothers are more involved if there is a supportive and cooperative relationship with the mother (Cutrona, Hessling, Bacon, & Russell, 1998), it is possibly due to a combination of these factors.

These findings must be interpreted with caution. The observations of mother–child free play were limited to just 3‐min, and therefore internal state language was coded within limited samples of speech in this study. However, given the size of the present sample and observations within the home, the nature of the data collection invariably results in a trade‐off with length of observations. Although mothers’ internal state language has been studied within similarly concentrated time‐periods (e.g., Paine et al., 2019; Roberts et al., 2013), longer natural observations of mother–child dyads in the home may give a more accurate reflection of children's daily conversational environments. Future studies would do well to investigate the impact of FNP on maternal language using longer samples of speech and unobtrusive methods by, for example, using Language Environment Analysis devices (LENA; Xu, Yapanel, & Gray, 2009), and by tracking the impact of FNP over multiple time points longitudinally. In addition, inclusion of appropriate measures of children's social understanding skills would enable researchers to examine effects on their understanding of the minds of themselves and others.

The present study provides a platform on which further investigations can explore ways in which mother–child interaction may be fostered by intensive home visiting interventions. Our investigation of family, maternal, and child factors associated with mothers’ internal state language highlights ways in which interventions may differentially impact subgroups; for example, mothers of boys or mothers not in education, employment, or training may benefit from interventions that focus on features of maternal speech. It is also essential that future studies investigate the impact of home visitation on other features of adolescent mothers’ language during mother–child interactions. The relationship between teenage motherhood and children's cognitive outcomes is mediated by maternal verbal stimulation, as measured by ratings of variety and complexity of a mothers’ vocabulary (Keown et al., 2001), as such, a wider investigation of maternal behaviors within mother–child interaction is an important next step in future research.

## CONFLICT OF INTEREST

The authors have no conflict of interest to declare.
